# Comorbidities complicating heart failure: changes over the last 15 years

**DOI:** 10.1007/s00392-022-02076-1

**Published:** 2022-08-17

**Authors:** Elles M. Screever, Martje H. L. van der Wal, Dirk J. van Veldhuisen, Tiny Jaarsma, Astrid Koops, Kuna S. van Dijk, Janke Warink-Riemersma, Jenifer E. Coster, B. Daan Westenbrink, Peter van der Meer, Rudolf A. de Boer, Wouter C. Meijers

**Affiliations:** 1 Department of Cardiology, Medical Center Groningen, University of Groningen, Antonius Deusinglaan 1, PO Box 30.001, 9700 RB Groningen, the Netherlands; 2 Department of Social and Welfare Studies, Linköping University, Linköping, Sweden

**Keywords:** Comorbidities, Heart failure, Hospitalization, Mortality, Obesity

## Abstract

**Aims:**

Management of comorbidities represents a critical step in optimal treatment of heart failure (HF) patients. However, minimal attention has been paid whether comorbidity burden and their prognostic value changes over time. Therefore, we examined the association between comorbidities and clinical outcomes in HF patients between 2002 and 2017.

**Methods and results:**

The 2002-HF cohort consisted of patients from The Coordinating Study Evaluating Outcomes of Advising and Counseling in Heart Failure (COACH) trial (*n* = 1,032). The 2017-HF cohort were outpatient HF patients enrolled after hospitalization for HF in a tertiary referral academic hospital (*n* = 382). Kaplan meier and cox regression analyses were used to assess the association of comorbidities with HF hospitalization and all-cause mortality.

Patients from the 2017-cohort were more likely to be classified as HF with preserved ejection fraction (24 vs 15%, *p* < 0.001), compared to patients from the 2002-cohort. Comorbidity burden was comparable between both cohorts (mean of 3.9 comorbidities per patient) and substantially increased with age. Higher comorbidity burden was significantly associated with a comparable increased risk for HF hospitalization and all-cause mortality (HR 1.12 [1.02–1.22] and HR 1.18 [1.05–1.32]), in the 2002- and 2017-cohort respectively. When assessing individual comorbidities, obesity yielded a statistically higher prognostic effect on outcome in the 2017-cohort compared to the 2002-HF cohort (*p* for interaction 0.026).

**Conclusion:**

Despite major advances in HF treatment over the past decades, comorbidity burden remains high in HF and influences outcome to a large extent. Obesity emerges as a prominent comorbidity, and efforts should be made for prevention and treatment.

**Graphical abstract:**

Created with BioRender.com.

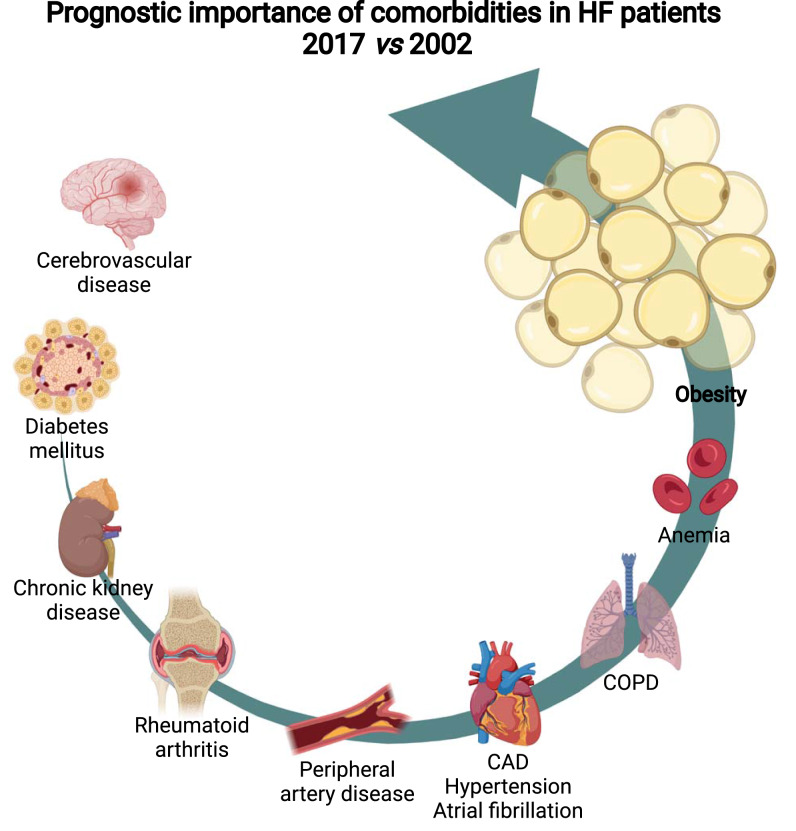

## Introduction

Multimorbidity, generally defined as the co-occurrence of more than one chronic condition, frequently accompanies heart failure (HF) [1, 2]. It has been put forward that comorbid conditions are important determinants of HF outcomes and have major impact on quality of life [3, 4]. In today’s ageing society, comorbidities are the rule rather than the exception [5, 6], making healthcare professionals face the challenge of simultaneous management of HF and underlying conditions. For years, the European Society of Cardiology (ESC) Heart Failure guidelines emphasize routine testing for coexisting comorbidities in all patients suspected for HF [7]. Also in the 2021 guidelines, assessment and treatment of comorbid conditions has a prominent position [8].

Despite advances in care and treatment modalities, HF patients – especially those with worsening and acute HF – remain at high risk for adverse events, with 1-year HF hospitalization and mortality rates up to 26 and 27%, respectively [9]. Because of the significant impact of comorbidities on outcome, we assessed whether prevalence and prognostic importance of comorbidities have changed over time in HF patients after an episode of acute decompensation requiring hospitalization.

## Methods

### 2002-HF cohort

The Coordinating Study Evaluating Outcomes of Advising and Counselling in Heart Failure (COACH) was a multicenter, randomized controlled trial in which 1023 patients were enrolled after hospitalization with acute HF as the primary diagnosis [10, 11]. Patients were included between October 2002 and February 2005 and randomly assigned to a control group (follow-up by a cardiologist) or one of two intervention groups (additional basic or intensive support by a nurse specialized in the management of HF patients). The mean follow-up period was 1.2 (± 0.5) years and patients were hospitalized for 13 days (± 10) after admission with acute HF. All patients were ≥ 18 years of age.

### 2017-HF cohort

This cohort consisted of all HF patients (*n* = 842) who visited the outpatient clinic of the University Medical Center Groningen (UMCG), in Groningen, the Netherlands, between March 2014 till December 2017. Their data were published previously [12].

In an attempt to make the 2002- and 2017-HF cohort as comparable as possible, patients from the 2017-HF cohort were only included if they visited the outpatient clinic on short term (i.e. < 2 weeks) after hospitalization for HF. In total, 382 HF patients were included in present analyses. Mean follow-up was 2.0 (± 1.0) years. All patients were ≥ 18 years of age and were treated according to the European Society of Cardiology (ESC) guidelines [7, 8].

### Establishing the diagnosis of heart failure

Regarding the 2002- and 2017-HF cohort, HF diagnosis was based on typical signs and symptoms of HF, for which hospitalization was required. HF hospitalization was defined as an unplanned overnight stay with acute HF as the primary diagnosis. In both cohorts, patients with reduced as well as preserved ejection fraction were included, according to the ESC HF guidelines in force at that time [7, 13﻿]. In present analyses, classification of patients into EF strata was based on current HF guidelines: HF with reduced EF (HFrEF, LVEF < 40%), HF with mildly reduced EF (HFmrEF, LVEF 40–49) and HF with preserved EF (HFpEF, LVEF ≥ 50).

### Ethics approval

Both studies conform to the 1975 Declaration of Helsinki; with regard to the 2017-HF cohort, the Medical Ethical Committee of the UMCG waived the need for informed consent due to the retrospective nature of the study. For the 2002-HF cohort (COACH), the Medical Ethical Committee of the UMCG gave approval and all study participants provided written informed consent.

### Definitions of comorbidities

The twelve cardiovascular (CV) and non-CV comorbidities included in the current study were coronary artery disease (CAD), atrial fibrillation/atrial flutter, hypertension, peripheral artery disease (PAD), cerebrovascular disease, anemia, obesity, hypercholesterolemia, diabetes mellitus, rheumatoid arthritis, chronic obstructive pulmonary disease (COPD) and chronic kidney disease (CKD). Comorbidity selection was based on available data from medical records from both HF cohorts. All medical data included in present analyses were adjudicated by one investigator and reviewed by a second investigator. Definitions of included comorbidities are shown in Supplemental Table 1. Besides the definitions, comorbidities were also included if they were noted in the inpatient or outpatient medical records or on the basis of appropriate medical treatment for this typical comorbidity. No additional diagnostic tests were performed to determine the presence of comorbidities.

**Table 1:** Baseline characteristics preceding incident clinical outcomes in the 2002- and 2017-HF cohort

Characteristics	2002-HF cohort *n* = 1,023	2017- HF cohort *n* = 382	*p*-value
Age (y), mean (SD)	71 (11)	70 (12)	0.11
Female sex, *n* (%)	384 (38)	150 (39)	0.55
SBP (mmHg), mean (SD)	118 (21)	117 (21)	0.19
DBP (mmHg), mean (SD)	68 (12)	69 (11)	0.49
BMI (kg/m^2^), mean (SD)	27 (5)	28 (6)	**0.016**
LVEF (%), mean (SD)	34 (14)	36 (14)	** < 0.001**
Cardiac device therapy, *n* (%)	80 (8)	106 (28)	** < 0.001**
HF subtype, n (%)			** < 0.001**
HFpEF	128 (15)	87 (24)	
HFmrEF	123 (15)	69 (19)	
HFrEF	581 (70)	207 (57)	
NYHA class, *n* (%)			** < 0.001**
I-II	513 (50)	219 (57)	
III-IV	495 (50)	163 (43)	
Comorbidities, *n* (%)			
Coronary artery disease	601 (59)	184 (48)	** < 0.001**
Hypertension	526 (51)	181 (47)	0.18
Atrial fibrillation/atrial flutter	509 (50)	196 (51)	0.60
Peripheral artery disease	168 (16)	69 (18)	0.47
Cerebrovascular disease	105 (10)	44 (12)	0.50
Anemia	270 (26)	172 (45)	** < 0.001**
Obesity	213 (23)	112 (29)	** < 0.001**
Hypercholesterolemia	400 (39)	28 (7)	** < 0.001**
Diabetes mellitus	289 (28)	193 (45)	** < 0.001**
Rheumatoid arthritis	67 (7)	10 (3)	**0.004**
Chronic obstructive pulmonary disease	268 (27)	99 (26)	0.91
Chronic kidney disease	610 (61)	232 (61)	0.71
Cancer	N/A	75 (20%)	N/A
Medication, *n* (%)			
ACEi/ARB	847 (83)	307 (80)	0.29
ARNI	N/A	1 (0.3)	N/A
β-blocker	677 (66)	356 (93)	** < 0.001**
Aldosterone antagonist	553 (54)	220 (58)	0.24
Diuretic	965 (94)	353 (92)	0.18
Biomarker levels, median [IQR]			
NT-proBNP (ng/L)	2528 [1289–5495]	1944 [957–3635]	** < 0.001**

N/A, data not applicable

Bold values denote statistical significance at the *p* < 0.05 level

*ACEi* angiotensin converting enzyme inhibitor, *ARB* angiotensin II receptor blocker, *ARNI* angiotensin receptor neprilysin inhibitor, *BMI* body mass index, *DBP* diastolic blood pressure, *HFpEF* heart failure with preserved ejection fraction, *HFmrEF* heart failure with mildly-reduced ejection fraction, *HFrEF* heart failure with reduced ejection fraction, *LVEF* left ventricular ejection fraction, *NT-proBNP* N-terminal pro-B-type natriuretic peptide, *NYHA* New York Heart Association, *SBP* systolic blood pressure

### Study endpoints

The primary endpoint was defined as the composite of hospitalization for HF and all-cause mortality (first to occur), during a total follow-up of 1.5 years. Hospitalization was defined as an unplanned overnight stay in the hospital due to worsening HF. Secondary endpoints were the separate components of the primary endpoint, namely HF hospitalization and all-cause mortality.

### Statistical analyses

Normally distributed data are presented as means ± SD. Non-normally distributed variables are presented as medians (interquartile range [IQR]). Categorical variables are presented as a number (%). Biomarker levels of NT-proBNP were log transformed prior to analysis to obtain approximately normal distributions. Differences between two groups were analyzed with the use of the Student’s T-test for normally distributed data, the Mann-Whitney U test for non-normally distributed data and the Spearman's chi square test for categorical variables. Differences in outcome in Kaplan-Meier analysis were compared using the log-rank test. In some of these analyses HF patients were categorized as having either 0–2, 3–4 or ≥ 5 comorbid conditions. To study the association between comorbidities and several endpoints, cox proportional hazard regression analysis was performed with comorbidities as a continuous variable. All analyses were adjusted for age and sex and a multivariate model (referred to as “clinical risk model”). This clinical risk model was based on the COACH risk engine - a valuable tool to predict survival and HF hospitalization in acute HF patients [14]. Since we used comorbidities as an independent variable, all comorbidities and factors what comorbidities are based on (i.e. blood pressure) were omitted from the COACH risk engine. Eventually, the clinical risk model used in present analyses consisted of the following parameters: age, sex, left ventricular EF (LVEF), serum sodium, plasma NT-proBNP and estimated glomerular filtration rate (eGFR). All reported *p* values are two-tailed. For the interaction term, a *p* value of < 0.10 was considered to indicate statistical significance. For all other analyses, a *p* value < 0.05 was considered statistically significant. Analyses were performed with STATA software version 16.0 (Stata Corp LP, College Station, TX, USA) and GraphPad Prism version 8.4.2 (GraphPad Software Inc., La Jolla, USA).

## Results

### Patient characteristics

Baseline characteristics of the 1,023 and 382 HF patients of the 2002- and 2017-cohort are presented in Table 1. 384 (38%) and 150 (39%) of the patients of the 2002- and 2017-cohort were female, patients from the 2017-cohort showed a slightly higher LVEF (36 *vs* 34%, *p* < 0.001), had lower rates of severe HF (NYHA III-IV) and lower levels of NT-proBNP (1944 *vs* 2528 ng/L, *p* < 0.001) compared to patients from the 2002-cohort. In the 2017-cohort, a higher percentage of patients could be classified as HF with preserved EF (HFpEF), compared to the 2002-HF cohort (24% *vs* 15%, *p* < 0.001) and a lower percentage as HF with reduced EF (HFrEF; 57% *vs* 70%, *p* < 0.001). At inclusion, patients from the 2017-HF cohort were more likely to have cardiac device therapy (28 *vs* 8%, *p* < 0.001) and to receive guideline-recommended therapies with β-blockers (93 *vs* 66%, *p* < 0.001) compared to patients from the 2002-cohort. No difference in medication use between both cohorts was seen in angiotensin converting enzyme inhibitors (ACEi)/angiotensin II receptor blockers (ARB), angiotensin receptor neprilysin inhibitors (ARNI), aldosterone antagonists and diuretics.

### Demographics of comorbidities

In the 2002- and 2017-HF cohort, almost all patients (e.g. 98% and 99%, respectively) suffered from comorbidities (Data not shown). The number of comorbidities ranged from 0 to 10 per individual, without difference in comorbidity burden between both HF cohorts (mean comorbidity burden of 3.9 in patients from both cohorts *p* = 0.92). Patients classified as HFpEF showed higher comorbidity burden compared to HFrEF patients (4.3 *vs* 3.7 comorbidities, *p* < 0.001).

**Fig. 1:**
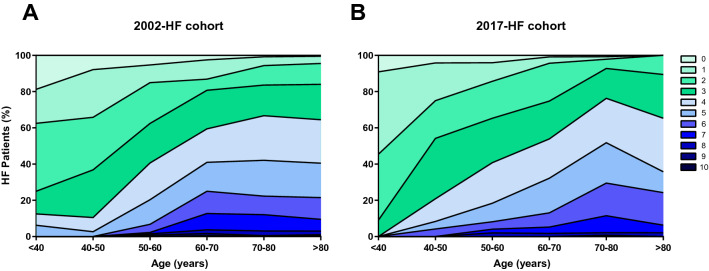
Distribution of comorbidities in different age categories in A) 2002-HF cohort and B) 2017-HF cohort. Increasing color intensity represents higher number of comorbidities as shown in the legend

To provide insight in the extensiveness of comorbidities present in HF patients, we plotted this according to age group (Fig. 1). In both HF populations, comorbidity burden substantially increased with age. While none of the patients younger than 40 years of age suffered from more than 4 comorbidities, this involved 40% and 39% of the patients aged 80 years and over, in the 2002- and 2017-cohort, respectively. HF patients from the 2002-cohort suffered more often from CAD (59 *vs* 48%, *p* < 0.001), hypercholesterolemia (39 *vs* 7%, *p* < 0.001) and rheumatoid arthritis (7 *vs* 3%, *p* = 0.004), while in patients from the 2017-cohort, anemia (45 vs 26%, *p* < 0.001), obesity (29 *vs* 23%, *p* < 0.001) and diabetes mellitus (45 *vs* 28%, *p* < 0.001) were more common (Table 1).

### Outcomes in 2002- and 2017-HF cohort

**Fig. 2:**
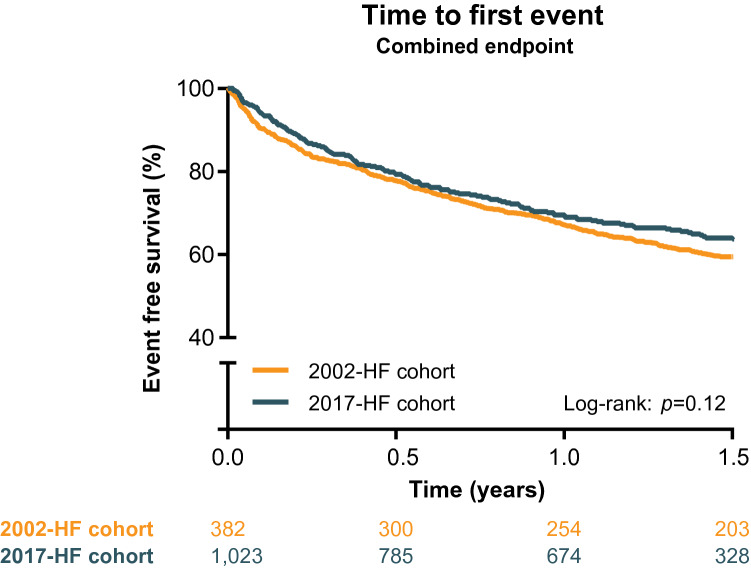
Kaplan-Meier curve for the combined endpoint (e.g. HF hospitalization and all-cause mortality), stratified by 2002- and 2017-HF cohort

During 1.5 years of follow-up, 260 patients of the 2002-cohort (25%) were readmitted for HF and 272 patients (27%) died, compared to 92 (24%) and 101 (26%) in the 2017-HF cohort. The primary composite endpoint was reached 411 (40%) and 138 (36%) times, respectively. No difference in event-free survival was observed between both HF cohorts (Log-rank *p* = 0.12) (Fig. 2).

To study if an increasing number of comorbidities was associated with a higher risk for the primary endpoint, HF patients were categorized as having either 0–2, 3–4 or ≥ 5 comorbid conditions. In both the 2002- and 2017-HF cohort, higher comorbidity burden was associated with higher risk for the primary combined endpoint (Log-rank for trend *p* < 0.0001 and *p* = 0.0037, respectively) (Supplemental Fig. 1A, B).

**Fig. 3:**
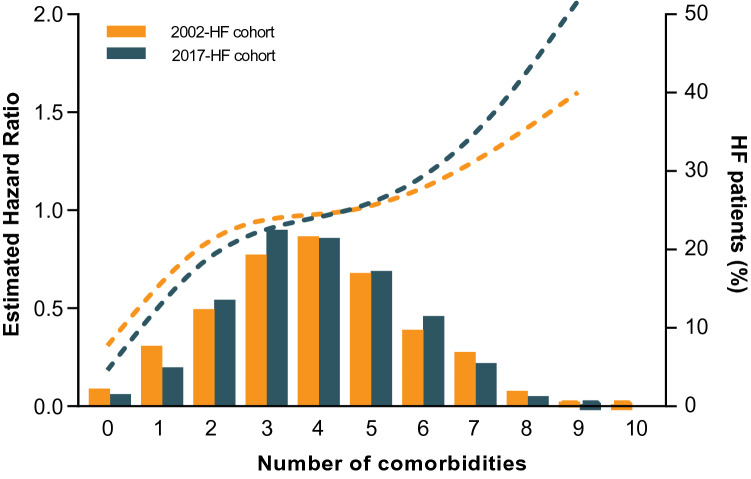
Relationship between number of comorbidities and the estimated hazard ratio of the combined endpoint (e.g. HF hospitalization and all-cause mortality) in patients from the 2002-HF cohort (orange) and the 2017-HF cohort (dark green). Data is adjusted for the clinical risk model: age, sex, LVEF, sodium, log(NT-proBNP) and eGFR. The histogram represents the percentage of patients with that specific number of comorbidities

Using cox proportional hazard regression analyses, we observed that comorbidities remained a significant predictor for the combined endpoint in both HF cohorts, even after adjustment for our clinical risk model (HR 1.12 [1.02–1.22], *p* = 0.017 and HR 1.18 [1.05–1.32], *p* = 0.006 for the 2002- and 2017-HF cohort respectively), without significant difference between both cohorts (*p* for interaction 0.840) (Table 2). This also applies to HF hospitalization and all-cause mortality separately (Table 2). Additionally, adjusted cox regression splines for the primary combined endpoint demonstrated the association between number of comorbidities and the primary combined endpoint for both HF cohorts (Fig. 3).

**Table 2:** Cox proportional hazard analyses for the risk on the combined endpoint (HF hospitalization and all-cause mortality) and HF hospitalization and all-cause mortality separately

2002-HF cohort				2017-HF cohort				
	HR	95% CI	*p*-value	Variables	HR	95% CI	*p*-value	*p*-value for interaction^b^
Combined endpoint				Combined endpoint				
Comorbidities	1.23	1.17–1.30	** < 0.0001**	Comorbidities	1.21	1.10–1.34	** < 0.0001**	
+ Age & Sex	1.20	1.14–1.27	** < 0.0001**	+ Age & Sex	1.20	1.08–1.34	**0.001**	
+ Clinical risk model^a^	1.12	1.02–1.22	**0.017**	+ Clinical risk model^a^	1.18	1.05–1.32	**0.006**	0.840
HF hospitalization				HF hospitalization				
Comorbidities	1.25	1.18–1.33	** < 0.0001**	Comorbidities	1.22	1.08–1.38	**0.002**	
+ Age & Sex	1.24	1.17–1.32	** < 0.0001**	+ Age & Sex	1.23	1.07–1.40	**0.003**	
+ Clinical risk model^a^	1.14	1.03–1.27	**0.015**	+ Clinical risk model^a^	1.18	1.02–1.36	**0.027**	0.900
All-cause mortality				All-cause mortality				
Comorbidities	1.25	1.18–1.33	** < 0.0001**	Comorbidities	1.22	1.07–1.38	**0.002**	
+ Age & Sex	1.21	1.14–1.29	** < 0.0001**	+ Age & Sex	1.18	1.04–1.35	**0.013**	
+ Clinical risk model^a^	1.12	1.00–1.25	**0.047**	+ Clinical risk model^a^	1.19	1.02–1.38	**0.022**	0.492

^a^Clinical risk model: age, sex, LVEF, sodium, log(NT-proBNP) and eGFR

^b^*p*-values for interaction refer to interaction term between 2002- and 2017-HF cohort

Bold values denote statistical significance at the *p* < 0.10 level for interaction terms and *p* < 0.05 level for all other analyses

*CI* conference interval, *HF* heart failure, *HR* hazard ratio

**Fig. 4:**
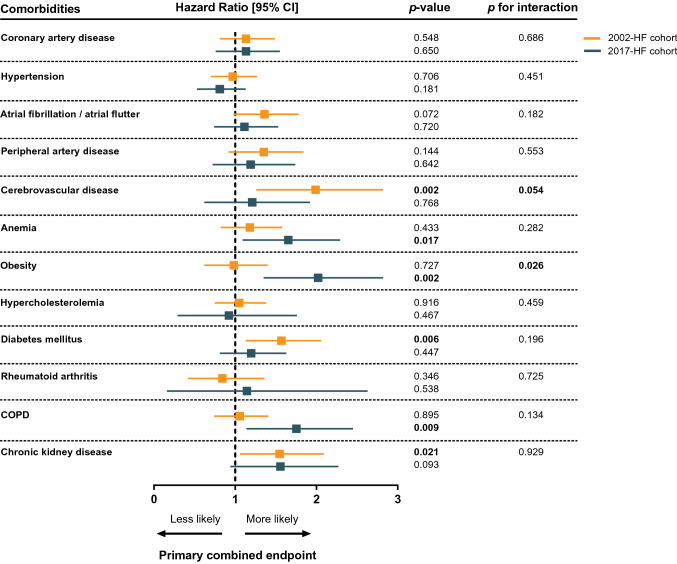
Forest plot showing the hazard ratio [95% CI] associated with individual comorbidities to the primary combined endpoint. Data is adjusted for the clinical risk model: age, sex, LVEF, sodium, log(NT-proBNP) and eGFR. *p*-values for interaction refer to interaction term between 2002- and 2017-HF cohort and respective comorbidity. Bold values denote statistical significance at the *p* < 0.10 level for interaction terms and *p* < 0.05 level for all other analyses

Given the awareness that individual comorbidities may have different impact on adverse outcomes, we used adjusted cox proportional hazard analyses to assess the association of individual comorbidities with the primary combined endpoint for both HF cohorts, as depicted in the Forest plot in Fig. 4. In the 2002-HF cohort, cerebrovascular disease, diabetes mellitus and CKD showed to be an independent predictor for adverse outcome, with respect to HF hospitalization and all-cause mortality (*p* = 0.002, *p* = 0.006, *p* = 0.021, respectively), and atrial fibrillation/flutter showed a trend towards poor outcome, although not significant. In the 2017-HF cohort, anemia, obesity and COPD were independently associated with an increased risk of the combined endpoint (*p* = 0.017, *p* = 0.002 and *p* = 0.009, respectively). Additionally, CKD showed a trend towards poor outcome, although not significant. CAD, hypertension, PAD, hypercholesterolemia and rheumatoid arthritis were not independently associated with primary outcome when correcting for the clinical risk model in both HF cohorts.

Test of interaction between individual comorbidities and HF-cohort (2002 or 2017) regarding clinical outcome is shown in the right column of Fig. 4. There was statistical evidence that the effect of cerebrovascular disease on outcome was greater in the 2002-cohort compared to the 2017-cohort (*p* for interaction 0.059). Interestingly, obesity yielded a stronger prognostic effect in the 2017-HF cohort compared to the 2002-HF cohort (*p* for interaction 0.026).

## Discussion

Overall, there are four major findings of this study: 1) our data confirm the persistent high burden of comorbidities in HF patients and the increase of comorbidity burden with age, 2) greater comorbidity burden was associated with higher rates of HF hospitalization and all-cause mortality after 1.5 years of follow-up, 3) individual comorbidities – and in particular obesity – have high prognostic value regarding outcome and 4) no differences in event-free survival were observed between the 2002- and 2017-HF cohort.

Firstly, we demonstrated that comorbidities were and are highly prevalent in HF patients and that comorbidity burden was higher as people aged. In recent years, multimorbidity is becoming increasingly common [15, 16], as has also been shown in a population-based study of 4 million UK residents [17]. In the present study, we observe similar comorbidity burden in HF patients over a time span of 15 years.

This highlights that comorbidities continue to play an important role in HF therapy and might implicate that medical treatment for HF patients is getting more and more complicated. Also, the substantial contribution of non-CV comorbidities emphasizes that treatment of HF patients should not only be approached from a cardiological perspective, but rather multidisciplinary, involving medical specialists as well as paramedics and nutritionists.

Although it is known that optimal medical therapy contributes to a substantial reduction in both HF morbidity and mortality [18], still many HF trials do not report comorbidities at baseline or during follow-up [19]. A systematic review of 118 HF trials concluded that assessment of comorbidities remains low and incomplete, which may be due to specific exclusion-criteria in efforts to avoid competing risks of mortality and to improve study drug tolerability [19]. This underscores the need for clinical trials specifically including HF patients with multimorbidity.

Whereas chronic comorbidity burden complicates optimal treatment of all patients [20], this appears to be especially profound in HF patients. In our study, the presence of comorbidities increased the relative risk for adverse outcomes with 12 and 18% in the 2002- and 2017-cohort, respectively. Additionally, increasing comorbidity burden was associated with higher risk for HF hospitalization and all-cause mortality. These results are in line with previously published data, including a cross-sectional study of over 122,000 individuals. Braunstein and colleagues showed that patients with multimorbidity (e.g. ≥ 5 comorbidities) accounted for more than 80% of total inpatient hospital days, while they make up only approximately 40% of the total HF patient population [3]. Also, an observational cohort study showed that female HF patients without major comorbidity – not males – have comparable survival estimates compared to age- and sex-matched control subjects [21]. Strikingly, in this study, the risk of comorbidities on outcome did not decrease in the past 15 years.

Individual comorbidities also contribute largely to adverse outcomes, and not only comorbidity burden per se. When comparing the 2002- with the 2017-HF cohort, a shift in comorbidity profile was observed. An interesting example is the prevalence of obesity: While in the 2002-cohort 23% of the patients could be classified as obese (BMI ≥ 30 kg/m^2^), this concerned 29% of the population of the 2017-cohort. This observation may be reflective of change in HF subtype: In the 2017-cohort, a higher percentage of patients were HFpEF patients compared to the 2002-cohort (24 *vs* 15%, respectively). Nevertheless, the prevalence of obesity is rising drastically, nowadays exceeding 20% in many European countries. It is even expected that half of the United States population will have obesity by 2030 [22].

Interestingly, in our study, the association between obesity and HF hospitalization and all-cause mortality was stronger in the 2017-cohort compared with the 2002-cohort, resulting in an 89% increased risk for adverse outcomes. These data might suggest a shift in prognostic importance of obesity in the last 15 years. Studies have repeatedly shown that obesity is associated with HF. Results from the Framingham Heart Study revealed that subjects with obesity had a two-fold increased risk for new-onset HF, compared to their lean counterparts [23]. A post-hoc analysis in 3,310 HFpEF patients from the TOPCAT trial assessed the impact of abdominal obesity on all-cause mortality, which showed a 52% increased risk for poor outcome [24]. On the other hand, it has been proven that reduction of obesity decreases the risk of HF. A prospective cohort study of Jamaly and colleagues showed that bariatric surgery decreased the risk of HF with 35% in Swedish obese subjects [25]. Several other studies emphasize the efficacy of exercise training and lifestyle adaptation to increase quality of life and prognosis in HF patients [26, 27]. Tackling obesity effectively – by physical activity programs and nutrition – will presumably also lead to a reduction in other comorbidities and eventually better outcome.

Lastly, we did not observe differences in event-free survival between the 2002- and 2017-HF cohort, although patients from the 2017-cohort showed lower levels of NT-proBNP and were more likely to receive guideline-recommended therapies with β-blockers and cardiac devices.

Lack of improvement in HF outcomes over the past years has been shown previously by an Australian study in over 12,000 patients hospitalized for HF. Over a period of ten years, no significant change was seen in 1-year all-cause mortality and all-cause rehospitalization rates. However, 1-year rehospitalization for HF decreased slightly during their study period (30% in 2005 *vs* 24% in 2014) [28]. Although HF guidelines emphasize assessment and treatment of comorbidities for years [7, 8], it is debatable what we exactly have achieved in recent years, regarding comorbidity therapy. With the high number of new-onset HF cases there is the urgent need for a different – most likely multidisciplinary – approach.

Collectively, our findings underscore the importance of comorbidities as a factor promoting exacerbation of HF and worsening survival. To the best of our knowledge, this is the first study to determine the association and prognostic value of overall and individual comorbidity burden in HF patients over a time span of 15 years.

## Strengths and limitations

In this retrospective study, two different HF cohorts were compared. A strength of this study is that patients from both study cohorts have been well-characterized upon cohort entry in a hospital specialized in HF care. From all patients demographic data, medical history, physical examination, laboratory measurements, electrocardiogram and echocardiogram were available.

However, there are several limitations to the present study that should be acknowledged. First, all analyses performed in this study were post-hoc analyses and should also be interpreted as such. Second, we have highlighted several major comorbidities in HF patients. Nevertheless, some comorbidities were not documented in one or both study cohorts, such as for example cancer, liver disease, mental disorders and cognitive impairment, which have been shown to be independent predictors of adverse outcomes in HF [3, 29–31]. For this reason, it was not possible to use a validated comorbidity score, for instance the Charlson or Elixhauser Comorbidity Index. However, we acknowledge that each comorbidity category may have an associated weight on outcome, as can also be seen in our own results. Third, comorbidities were only included at the time of cohort entry, but were not examined during the follow-up period. Severity of comorbidities at baseline and disease progression over time (e.g. glycemic control in diabetes mellitus patients) may also have a great impact on outcome, rather than the exact number of comorbidities alone. Furthermore, novel treatment strategies for HF and associated comorbidities have been emerging. This is evident from the higher proportion of patients receiving cardiac device therapy and β-blockers in the 2017-HF cohort in comparison to the 2002-HF cohort. Nevertheless, only 1 patient (0.3%) from the 2017-HF cohort was treated with ARNI at inclusion, which may not be fully reflective of the contemporary HF population in 2022. Medication use was only described at the time of cohort entry, which might explain the low percentage of patients using ARNI in the 2017-HF cohort. Additionally, large registries describe that adaption of ARNI in medical practise was a slow process with only 2% of patients using ARNI one year after FDA approval [32]. Lastly, a large proportion of HF patients in the 2017-HF cohort were not eligible for ARNI therapy based on their LVEF classification.

Last, we did not observe differences in overall survival between the 2002- and 2017-HF cohort. This might be explained by differences in both HF populations. The 2002-HF cohort was a multicenter design with specific enrollment criteria, in which intervention was mainly aimed at education and support, rather than medical treatment. By contrast, the 2017-HF cohort was a single-center study in which all patients were included in an academic center specialized in HF care. Consequently, it is possible that the 2017-cohort represented more complex HF patients in comparison with the 2002-HF cohort, which has consequences on outcome. Furthermore, both cohorts consist of post-discharge patients, who are known to be at higher risk of adverse outcomes.

## Conclusion

Comorbidities are daily practice in HF and determine HF outcome to a large extent. In this study, we validate the strong impact of comorbidities – and in particular obesity – on prognosis and conclude that comorbidity burden in HF patients has not improved in recent years, despite major advances in HF treatment. These data suggest that a multidisciplinary approach – rather than the “cardiological” approach – is strongly needed to optimally treat HF patients. Since obesity directly contributes to CV risk factors and onset of HF, further studies to determine the effect of lifestyle adaptation programs, physical activity and nutritional status are urgently needed.
